# Author’s reply to the Letter to the Editor “The study of olfactory dysfunction in SARS-CoV-2 variants”

**DOI:** 10.1007/s00405-022-07569-3

**Published:** 2022-07-29

**Authors:** Constantin A. Hintschich, Veronika Vielsmeier, Christopher Bohr, Jan Hagemann, Ludger Klimek

**Affiliations:** 1 Department of Otorhinolaryngology, Regensburg University Hospital, Franz-Josef-Strauss-Allee 11, 93053 Regensburg, Germany; 2 Department of Otorhinolaryngology, Mainz University Hospital, Mainz, Germany; 3 Center for Rhinology and Allergology, Wiesbaden, Germany

Dear editor,

We are delighted by the interest in our publication “Prevalence of acute olfactory dysfunction differs between variants of SARS-CoV-2—results from chemosensitive testing in wild type, VOC alpha (B.1.1.7) and VOC delta (B.1617.2)” and want to thank Lechien et al*.* for their kind estimation and the thoughtful discussion of our manuscript [1].

In this study we psychophysically confirmed a lower prevalence of olfactory dysfunction in the SARS-CoV-2 variants of concern (VOCs) alpha and delta compared to the wild-type. This is in accordance with recent publications: Coelho et al*.* showed odds ratios of 0.50, 0.44 for patients’ self-ratings of olfactory dysfunction in VOCs alpha and delta compared to the wild-type [2]. Similarly, Klimek et al*.* found a significantly higher TDI score in patients with VOC delta compared to wild-type [3].

Our data has been collected under the challenges of acute COVID-19. Hence, as addressed by Lechien et al*.* our study has some limitations:

Chemosensitive assessment is normally performed in specialized departments and comprises the collection of the patient’s history, a clinical examination including nasal endoscopy and psychophysical testing. As SARS-CoV-2 positive patients are normally quarantined, home-based approaches have been established using self-prepared or shipped test kits combined with questionnaires [4, 5], online surveys [6], or telephone interviews [7].

I. Besides remote olfactory testing we recorded preconditions of both general health and chemosenses and the individual course of the SARS-CoV-2 infection. Patients with a previously known hyposmia or related conditions such as rhinosinusitis, traumatic brain injury or neurological diseases were excluded to minimize a possible bias of the results. However, due to the strict word limitation of the Short Communication, we could unfortunately not describe the methods in full detail and not present all findings of our study.

II. We do completely agree that testing of threshold, discrimination, and gustation (TDI, Sniffin’ Sticks) remains the gold standard for the psychophysical assessment of olfaction [8]. Due to the special circumstances of home-quarantine during acute COVID-19 this was hardly possible. Hence, we chose the well-established 8-item NHANES pocket smell test and the 16-item identification test to assesses olfaction in a remote approach. Different than stated by Lechien et al*.* a cut-off values have been established for both the 8-item NHANES pocket smell test (hyposmia: five or less correct answers of eight) [9] and the 16-item identification (hyposmia: eleven or less correct answers of 16) [10]. Therefore, the cut-off value for normosmia is 75% for both used tests.

III. The self-assessment of Sniffin’ Sticks might be prone to bias for various reasons as stated correctly by Lechien et al. To ensure the correct execution patients were instructed through an established telemedicine approach [7]. Moreover, the very same 16-item identification test [11] as well as other smell tests [12] have been previously validated for self-administration.

Recently, evidence of a declined lower prevalence of patients self-rated olfactory disorders in VOC omicron has been published [2, 13, 14] and the psychophysical data will likely follow. However, our study remains unique to psychophysically compare wild-type with the VOCs alpha and delta.

## References

[1] Hintschich CA (2022). Prevalence of acute olfactory dysfunction differs between variants of SARS-CoV-2-results from chemosensitive testing in wild type, VOC alpha (B.1.1.7) and VOC delta (B.1617.2). Eur Arch Otorhinolaryngol.

[2] Coelho DH (2022). Decreasing incidence of chemosensory changes by COVID-19 variant. Otolaryngol Head Neck Surg.

[3] Klimek L (2022). Olfactory dysfunction is more severe in wild-type SARS-CoV-2 infection than in the Delta variant (B.1.617.2). World Allergy Organ J.

[4] Hintschich CA (2022). Gustatory function in acute COVID-19—results from home-based psychophysical testing. Laryngoscope.

[5] Hintschich CA (2022). Persisting olfactory dysfunction in post-COVID-19 is associated with gustatory impairment: results from chemosensitive testing eight months after the acute infection. PLoS ONE.

[6] Hintschich CA (2020). Psychophysical tests reveal impaired olfaction but preserved gustation in COVID-19 patients. Int Forum Allergy Rhinol.

[7] Klimek L (2021). Telemedicine allows quantitative measuring of olfactory dysfunction in COVID-19. Allergy.

[8] Hummel T (1997). ‘Sniffin’ sticks': olfactory performance assessed by the combined testing of odor identification, odor discrimination and olfactory threshold. Chem Senses.

[9] Rawal S (2015). The taste and smell protocol in the 2011–2014 US National Health and Nutrition Examination Survey (NHANES): test-retest reliability and validity testing. Chemosens Percept.

[10] Oleszkiewicz A (2019). Updated Sniffin' Sticks normative data based on an extended sample of 9139 subjects. Eur Arch Otorhinolaryngol.

[11] Mueller CA (2006). A self-administered odor identification test procedure using the "Sniffin' Sticks". Chem Senses.

[12] Vaira LA (2020). Validation of a self-administered olfactory and gustatory test for the remotely evaluation of COVID-19 patients in home quarantine. Head Neck.

[13] Boscolo-Rizzo P (2019). Coronavirus disease 2019 (COVID-19)-related smell and taste impairment with widespread diffusion of severe acute respiratory syndrome-coronavirus-2 (SARS-CoV-2) Omicron variant. Int Forum Allergy Rhinol.

[14] Menni C (2022). Symptom prevalence, duration, and risk of hospital admission in individuals infected with SARS-CoV-2 during periods of omicron and delta variant dominance: a prospective observational study from the ZOE COVID Study. Lancet.

